# Systematic Review of the Treatment of Persistent Hyperparathyroidism Following Kidney Transplantation

**DOI:** 10.3390/biomedicines11010025

**Published:** 2022-12-22

**Authors:** Miłosz Miedziaszczyk, Katarzyna Lacka, Oskar Tomczak, Aleksander Bajon, Marta Primke, Ilona Idasiak-Piechocka

**Affiliations:** 1 Department of Nephrology, Transplantology and Internal Medicine, Poznan University of Medical Sciences, 60-355 Poznan, Poland; 2 Department of Endocrinology, Metabolism and Internal Medicine, Poznan University of Medical Sciences, 60-355 Poznan, Poland; 3 1st Department of Anesthesiology and Intensive Therapy, Poznan University of Medical Sciences, 61-848 Poznan, Poland; 4 Student Scientific Section of Department of Nephrology, Transplantology and Internal Medicine, Poznan University of Medical Sciences, 60-355 Poznan, Poland; 5 Student’s Scientific Section of Department of Endocrinology, Metabolism and Internal Medicine, Poznan University of Medical Sciences, 60-355 Poznan, Poland

**Keywords:** persistent hyperparathyroidism, kidney transplantation, renal transplantation, treatment, systematic review

## Abstract

Chronic kidney disease–mineral and bone disorder is one of the complications associated with chronic kidney disease. About 10–50% of patients following kidney transplantation have persistent hyperparathyroidism. Hypercalcaemic hyperparathyroidism has a negative impact on the kidney transplant outcome; therefore, it requires treatment. The data regarding the treatment of persistent hyperparathyroidism provided in scientific publications are divergent and contradictory. Therefore, the aim of our systematic review was to evaluate the efficacy of persistent hyperparathyroidism treatment in patients following kidney transplantation. The Cochrane, PubMed, and Scopus databases were browsed independently by two authors. The search strategy included controlled vocabulary and keywords. The effectiveness of calcitriol, paricalcitol, cinacalcet, and parathyroidectomy was compared and analysed. The mean calcium and parathormone (PTH) concentrations per patient in the group of paricalcitol increased by 1.27% and decreased by 35.14% (n = 248); in the group of cinacalcet decreased by 12.09% and 32.16% (n = 368); and in the group of parathyroidectomy decreased by 19.06% and 86.49% (n = 15) at the end of the study compared to the baseline (n = 244, n = 342 and n = 15), respectively. Paricalcitol, cinacalcet, and parathyroidectomy decreased the intact PTH level. Cinacalcet and parathyroidectomy lowered calcium levels in renal transplant patients with hypercalcaemia. Conversely, paricalcitol increased the serum calcium concentration. Cinacalcet seems to be a good candidate in the treatment of post-transplant hyperparathyroidism.

## 1. Introduction

Secondary hyperparathyroidism (SHPT) is a condition in which the parathormone (PTH) level is elevated to compensate for the calcium–phosphorus imbalance. An increase in PTH secretion is triggered by hypocalcaemia, hyperphosphataemia, or a decreased active vitamin D [1]. In pre-kidney transplantation (pre-KTx) patients with advanced chronic kidney disease (CKD), calcitriol (active vitamin D) deficiency is related to the inability to convert vitamin D to its physiologically active form, 1,25-cholecalciferol. Subsequently, it causes a decrease in intestinal absorption of calcium and a low serum calcium level [2]. Furthermore, a decreasing glomerular filtration rate (GFR) in CKD leads to reduced phosphate clearance and hyperphosphataemia [3]. In turn, excess serum phosphorus increases PTH secretion, as well as results in hypocalcaemia (phosphorus forms complexes with calcium) and stimulates fibroblast growth factor-23 (FGF-23) [4]. SHPT is one of the most common complications associated with CKD. Mineral metabolism imbalances increase the risk of bone fractures, renal osteodystrophy, and vascular calcification. This, in turn, contributes to the development of cardiovascular diseases and results in higher mortality rates among CKD patients [5]. Moreover, prolonged exposure to the aforementioned electrolyte disturbance leads to hyperplasia of parathyroid tissue, which persists even after kidney transplantation [2]. One of the accompanying conditions related to CKD is SHPT, which may remain problematic post-transplantation [6]. The incidence of persistent hyperparathyroidism after kidney transplantation is estimated to be 10–50%; it depends on the age group and increases with age [7,8,9]. There is no consensus regarding a PTH level which clearly defines the presence of persistent post-transplant hyperparathyroidism. Nevertheless, most clinicians consider a PTH level greater than two times normal (>130 pg/mL) to be consistent with persistent post-transplant hyperparathyroidism (PT-HPT) within 12 months post-transplant [6]. SHPT initially manifests as polyclonal diffuse hyperplasia, which then evolves into multiple monoclonal nodules. Lesion advancement correlates with the increasing weight of the parathyroid gland, although even in low-weight parathyroid glands nodular lesions have been detected [10]. Taniguchi et al. [11] reported that calcium-sensing receptors, vitamin D receptors, as well as apoptosis increased following transplantation with diffuse hyperplasia; therefore, shrinkage of this part is likely to be observed. This is associated with a gradual decrease of PTH levels after transplantation observed in some patients [12,13]. The remaining nodular hyperplastic part of the parathyroid gland does not demonstrate similar receptor changes, thus indicating that they account for most tertiary HPT effects [11]. Studies showed that the most significant independent risk factors of post-transplant HPT include high pre-transplantation levels of PTH and glomerular filtration rate of the kidney transplant. Other, such as long-term duration of dialysis, large maximum parathyroid gland size, high (<2 weeks) Ca level, and high post-transplant (<2 weeks) ALP concentration contribute to the exacerbation of this abnormality. Thus, early control of risk factors could positively affect post-transplant outcomes [12,13,14,15]. Additionally, after the transplantation, suboptimal graft function, immunosuppressive drugs, metabolic acidosis, hyperphosphataemia, and low vitamin D levels also may increase PTH secretion and contribute to persistent HPT [16]. Moreover, HPT associated with the deterioration of renal transplant function is also of note, when PTH levels increase along with the deterioration of renal transplant function to maintain normal phosphataemia and normocalcaemia [17]. Persistent hypercalcaemia in post-transplant patients plays a role in the early calcification of renal allografts [18] and progression of artery calcification [19]. In transplantation patients, unlike the dialysis patients, an increased PTH level is accompanied by hypophosphataemia. In fact, these two factors, i.e., high PTH and low phosphate, may affect bone metabolism differently. An elevated PTH level seems to have a protective effect on osteoblasts by means of preserving their survival. In contrast, a decreased phosphorus level has been associated with impaired osteoblastogenesis and early osteoblast apoptosis. Therefore, the abovementioned divergent effects may result in low bone turnover [20]. Despite mineral balance abnormalities, SHPT may lead to other systemic problems. Ivarsson et al. [21] reported that increased PTH was associated with new-onset diabetes mellitus after transplantation, which has a negative impact on both the graft and patient survival.

There is an ongoing debate with regard to the early treatment of persistent post-transplant hyperparathyroidism. On the one hand, some researchers emphasise that this condition often improves with time. However, other studies provide data indicating that improvement in parathyroid function at 3 months post-transplant is unlikely [7]. Another argument is that the withdrawal of some medications, such as cinacalcet, at the time of renal transplantation does not constitute a risk factor for allograft calcifications in the early post-transplantation period [22]. Nevertheless, hypercalcaemic hyperparathyroidism itself has a negative impact on the kidney transplant outcome; therefore, it requires treatment. Furthermore, the first year of the post-transplantation period is essential in terms of bone loss [23]. In addition to its role in bone disease, elevated PTH level is a risk factor of vascular calcification, which leads to an increased post-transplant cardiovascular morbidity and mortality [24].

## 2. Materials and Methods

The aim of our systematic review was to evaluate the efficacy of persistent hyperparathyroidism treatment in patients following kidney transplantation (KTx).

**Figure 1:**
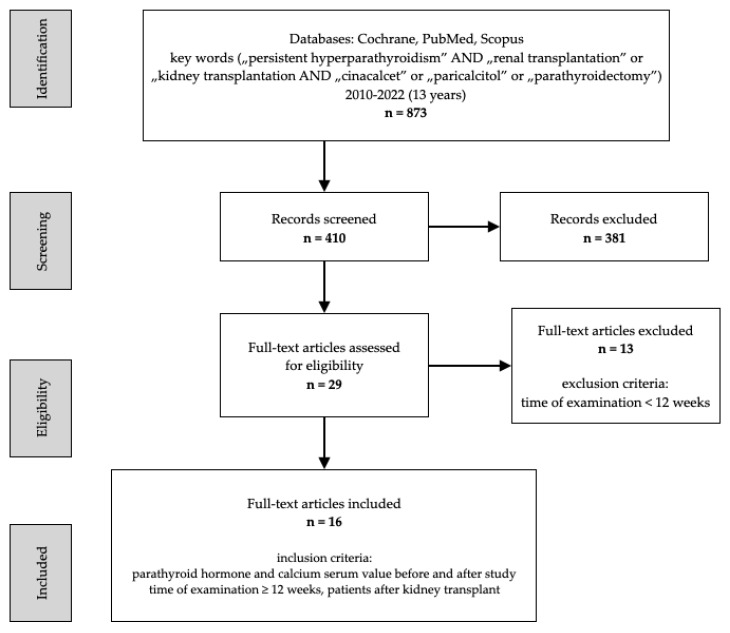
Stages of including articles in the review, PRISMA flowchart.

The search strategy included controlled vocabulary and keywords. The Cochrane, PubMed, and Scopus databases were browsed independently by two authors. The main search criteria were ‘persistent hyperparathyroidism’ AND ‘renal transplantation’ or ‘kidney transplantation’ AND ‘cinacalcet’ or ‘paricalcitol’ or ‘parathyroidectomy’. Studies published in the past 13 years were considered for this review (Figure 1). The PRISMA Checklist was verified during the draft of the review. A flowchart of the systematic literature search was developed according to PRISMA guidelines. Study protocol has been registered (ID CRD42022369857). The papers were selected depending on their clinical importance and Greenhalgh’s evidence hierarchy. Therefore, case reports were considered as having low clinical importance; nevertheless, they were mentioned in our review in order to provide a broader perspective on the topic. Data were collected, arranged, and subsequently the selected information was depicted in Table 1 and Table 2. Nevertheless, due to no data on the topic, it was impossible to include the presence of primary hyperparathyroidism as an exclusion criterion. Additionally, we could not provide the number of treated SHPT patients and the number of the remaining HPT cases. Statistical calculations were performed using the direct method of arithmetic mean of grouped data.

Studies [37,38] represent the same group of patients; the difference is in the duration of the study.

## 3. Results

The mean calcium concentration per patient in the paricalcitol efficacy studies increased by 1.27% (n = 248) at the end of the study compared to the baseline (n = 244). The mean intact PTH (iPTH) concentration per patient decreased by 35.14% (n = 248) at the end of the study compared to the baseline (n = 244). The mean calcium concentration per patient in the cinacalcet efficacy studies decreased by 12.09% (n = 368) at the end of the study (n = 342). The mean iPTH concentration per patient decreased by 32.16% (n = 368) at the end of the study compared to the baseline (n = 342). The mean calcium concentration per patient in the parathyroidectomy efficacy studies decreased by 19.06% (n = 15) at the end of the study compared to the baseline (n = 15). The mean PTH concentration per patient decreased by 86.49% (n = 15) at the end of the study compared to the baseline (n = 15).

### 3.1. Calcitriol

Calcitriol is an active form of cholecalciferol (vitamin D3). The synthesis of calcitriol requires two hydroxylations. The first takes place in the liver and results in the production of 25-hydroxycholecalciferol (25-hydroxyvitamin D3, calcifediol, 25(OH)D3) from cholecalciferol. Thereafter, due to the action of 1α-hydroxylase in the kidneys, 1α,25-dihydroxyvitamin D3 (calcitriol, 1,25(OH)2D3) is synthesised. Factors which stimulate this process include parathyroid hormone (PTH), low calcium, and low phosphate levels. Conversely, high levels of fibroblast growth factor 23 (FGF23) and phosphate cause a reduction in the synthesis of calcitriol. An active form of vitamin D3 (calcitriol) binds to vitamin D receptor (VDR), which leads to the stimulation of renal and intestinal absorption of calcium and phosphate, as well as a downregulation of PTH synthesis. Additionally, calcitriol has a direct impact on osteoclasts and osteoblasts; it also modulates immune and inflammatory response, as well as renin production [39,40].

Six months after a renal transplant, as a result of lower FGF23 levels, levels of serum calcitriol increase significantly [41,42]. However, immunosuppressive therapy contributes to a decrease in 1,25(OH)2D3. Moreover, glucocorticoids intensify vitamin D catabolism and promote an increase of PTH levels. Among kidney transplant recipients, reduced 25(OH)D levels (ercalcifediol, 25(OH)D2 and 25(OH)D3), as well as a deranged metabolism of vitamin D3 are highly prevalent, which contributes to the presence of chronic kidney disease–mineral and bone disorder (CKD-MBD). Precise mechanisms of alterations in calcitriol metabolism are still insufficiently understood [39]. Several trials considered calcitriol therapy among patients with persistent secondary hyperparathyroidism (SHPT) following renal transplant. According to Tillmann et al. [43], the application of vitamin D therapy, in this clinical situation, is limited. Patients with persistent SHPT frequently suffer from hypercalcaemia, while SHPT (prior to the renal transplant) co-occurs with normocalcaemia or hypocalcaemia. Conversely, calcitriol supplementation reduces PTH levels [44,45] and protects against loss of bone mineral density [40,45]. In fact, Wada et al. [45] observed that a combination of denosumab and calcitriol enabled effective treatment of a patient with persistent SHPT and a massive bone loss. Denosumab in monotherapy resulted in hypocalcaemia, which was effectively improved by calcitriol supplementation. Nonetheless, further research on this treatment method should be conducted. Vitamin D also exhibits a protective impact on the cardiovascular system, and may play a role in the treatment of proteinuria, which is particularly important for patients with CKD [39]. Another application of an active form of vitamin D, combined with calcium supplementation, is in patients following parathyroidectomy, as a form of treatment for persistent SHPT. The studies indicate that it prevents hypocalcaemia, caused by the hungry bone syndrome, which may appear after resection [42]. Nevertheless, due to the scarcity of large clinical trials, an appropriate dose of calcitriol and a precise mode of action remain unknown. There is a great need for further investigation and confirmation of its application in the treatment of persistent SHPT [44].

### 3.2. Paricalcitiol

Paricalcitiol (19-nor-1,25-dihydroxyvitamin D2) is a synthetic vitamin D analogue which binds to the vitamin D receptors in a similar manner as 1,25-dihydroxyvitamin D3 (calcitriol). Therefore, by affecting the retinoid X receptor, it regulates the transcriptional activity of vitamin D-responsive genes. The crucial result of that action is a decrease in the secretion of parathyroid hormone (PTH) in a dose-dependent manner, which suppresses parathyroid hyperplasia [46]. According to the available sources, the paricalcitol decreasing effect on PTH is undoubted [25,26,27,28,47,48,49,50]. However, information about the spectrum of action of this medication may differ depending on the specific source. Žilinská et al. [43] reported an increased calcaemia and calciuria, accompanied by diminishing phosphorus levels after paricalcitol treatment. Nevertheless, no case of hypercalcaemia was recorded, and the calcium plasma levels were within the reference range. On the other hand, other studies did not demonstrate any significant fluctuation in calcium blood levels [26,49]. However, bone density measurements and radiographic evaluation following paricalcitol administration showed significant improvement in bone condition [27,47,49], whereas serum bone-specific alkaline phosphatase and osteocalcin decreased as compared to baseline [25,27].

The abovementioned paricalcitol effect on calcium–phosphorus balance and bone turnover may be the result of changes on the genetic level, as well as on inner-cell signalling pathways [48,50]. Therefore, KLOTHO was investigated. This multifunctional protein functions as a co-receptor for fibroblast growth factor-23 (FGF23) to modulate FGF23 signal transduction. The putative physiologic role of FGF23 is to act as a counter-regulatory phosphaturic hormone to maintain phosphate homeostasis in response to vitamin D [50]. Donate-Correa et al. [48] investigated the ratio of the unmethylated/methylated klotho promoter DNA, which did not change in controls, although it was increased by 177% in paricalcitol-treated subjects. The increase in this ratio was independently associated with the change in messenger RNA expression levels, which showed a significant and direct correlation with an elevation of serum klotho concentrations. Subsequently, a significant increase in the serum levels of FGF-23 after paricalcitol administration was detected. Another pleiotropic effect of paricalcitol worth bearing in mind is a significant reduction of C-reactive protein (CRP) serum concentrations. CRP is a best-characterized biomarker of inflammation; thus, reducing its level is a sign of alleviating fibrosis and other inflammation-associated adverse effects on the graft [25,28,48,49]. The majority of tests approved the anti-proteinuric effect of paricalcitol; hence, implying its protective properties [27,28,47,51].

In contrast, Pihlstrøm et al. [25] observed no significant reduction/prevention of albuminuria, nor any influence on allograft gene expression. No significant group differences were found in the development of fibrosis, or chronic allograft damage index between the control and treated groups. Finally, only one study provided data regarding significant changes in the urinary peptidome of kidney transplant patients treated with paricalcitiol. However, these changes were not unidirectional, and some peptide levels were observed to increase, whereas others decreased [49]. According to the literature, the proven safety of paricalcitol legitimises its common use. Despite the possible occurrence of side effects, this drug is well-tolerated and it is considered to be safer than other alternatives [52,53].

### 3.3. Cinacalcet

The subtotal parathyroidectomy was the standard treatment of persistent hyperparathyroidism with hypercalcaemia in kidney transplant recipients. Currently, it has been replaced by the calcimimetic cinacalcet. In their publication, Soliman et al. aimed to prove the advantage of surgery over pharmacotherapy; however, no difference was found between the two groups in terms of the analysed parameters [54]. Furthermore, Mogl et al. compared the results of parathyroidectomy performed in patients receiving cinacalcet and those who were not treated with this drug. No difference was observed in the recurrence, or persistence of hyperparathyroidism, surgery duration, hospitalisation, or complications rate [55]. Calcimimetic agents represent a therapeutic alternative in transplant patients with persistent hyperparathyroidism and hypercalcaemia. They inhibit parathyroid hormone (PTH) secretion, increasing the sensitivity of the calcium-sensitive receptor in the parathyroid gland [56]. Some studies suggest this mechanism may be independent of PTH serum level suppression. The increase in bone-specific alkaline phosphatases, biochemical markers of bone accretion and a significant decrease in fasting urine calcium suggest the possibility of a beneficial impact of cinacalcet on bone remodelling [57]. The studies conducted by Pinho et al. demonstrated that all patients exhibited a significant reduction in parathyroid hormone levels iPTH from a mean value of 242.04 ±105.82 pg/mL to a mean value of 145.62 ± 54.99 pg/mL. In addition, serum calcium levels normalized during the study period, from 11.16 mg/d to 9.95 mg/dL [29]. Vaquero et al., in their paper referring to the effect of cinacalcet on iPTH serum levels, showed that the drug administrated once daily reduced PTH in renal transplant recipients without holding PTH for 24 h. They noticed a progressive increase to a similar level to baseline 24 h after the administration [58]. Following a discontinuation of cinacalcet, calcaemia persisted at normal levels in 50%, although the drug had to be reintroduced in the other 50% after 10 ± 7.9 months. Nonetheless, there are currently no indicators suggesting in which group of patients further treatment may be discontinues [59]. As pointed out by Tillmann et al., the dysfunction of the parathyroid glands before the transplantation is associated with a clinically significant hyperparathyroidism after surgery. According to their study, PTH levels at 4 weeks post-transplantation may serve as a marker for the occurrence of hypercalcemic hyperparathyroidism during the follow-up [43]. Discontinuing cinacalcet within the first month following kidney transplantation frequently results in hypercalcaemia [60], although it is not a risk factor for allograft calcifications in the early post-transplant period. According to Paschoalin et al., allograft calcification was found in serial protocol biopsies after transplantation among patients receiving cinacalcet on dialysis, which was discontinued after the surgery. In fact, all biopsies showed nephrocalcinosis: either intratubular calcifications or calcifications in the parenchyma [22]. Furthermore, in patients with normocalcaemic persistent secondary hyperparathyroidism after kidney transplantation, cinacalcet improved the control of serum PTH without causing calcaemia, or phosphataemia. Observations were conducted on a group of 32 patients. Treatment with cinacalcet began after 16 months following KTx (median dose of 30 mg/day). Levels of iPTH decreased from a median of 364 pg/mL at the start of the study to 187 after 6 months (48.6% reduction) and to 145 after 12 months (60.2% reduction), without any changes in calcium and phosphorus [30]. Niel et al. in their study suggested that cinacalcet could be used in a paediatric population with an effect comparable to that observed in adults. The authors reported a case of a child after bilateral nephrectomy due to nephroblastoma. After renal transplantation, ionized serum calcium levels remained increased (1.6 mmol/L, 6.4 mg/dL), iPTH levels were high (348 ng/L), whereas serum phosphorus levels were low (1.24 mmol/L, 3.83 mg/dL). At that time, the patient received phosphate salts. A renal biopsy revealed traces of calcium deposits, and the ultrasonography showed a 9 mm × 4 mm parathyroid adenoma. Cinacalcet was introduced 4 months following renal transplantation; the initial dose was 15 mg/day, increased every 15 days up to 60 mg/day. At 7 months after renal transplantation, total serum calcium levels were normal (2.37 mmol/L, 9.48 mg/dL), as well as ionized serum calcium levels (1.24 mmol/L, 4.96 mg/dL) and serum phosphorus (1.42 mmol/L, 4.39 mg/dL); additionally, iPTH decreased (294 ng/L). A second renal biopsy performed as a control showed no traces of calcium deposits. Cervical ultrasonography performed 9 months after renal transplantation demonstrated no visible parathyroid hypertrophy, which was confirmed by another ultrasonography performed 6 months later [61]. In the study by Torregros et al. including 193 subjects (n = 193), a long-term effect of cinacalcet was verified. It was a retrospective study based on data from 17 renal transplant units from Spain. After cinacalcet pharmacotherapy mean calcium levels and iPTH decreased, whereas the mean phosphorus level was increased. The effects were maintained for up to 3 years. No changes were observed in the renal function or calcineurin inhibitor levels. Only 4.1% of patients discontinued cinacalcet due to intolerance, and 1.0% due to the lack of efficacy [31]. Zavvos et al. made similar observations based on a 5-years-long follow-up performed on a group of 47 patients. Adverse reactions were observed in four patients, comprising mild gastrointestinal complaints [32]. Satisfactory results of the therapy over 53 ± 7.4 months are also described by Paschoalin et al. [33]. There was no statistical difference in the percentage change in bone mineral density (BMD) at the femoral neck between the cinacalcet and placebo groups [34]. However, the issue emerged as to whether patients with hyperparathyroidism who received cinacalcet could be at increased risk of renal calcium deposits due to hypercalciuria and a subsequent renal transplant dysfunction. Seager et al. in their article described the first well-documented case in which cinacalcet contributed to the development of new renal calculi in a post-transplant patient with hyperparathyroidism (iPTH 346 pg/mL), hypercalcaemia (11.3 mg/dL), and good renal function (1.45 mg/dL). Interval imaging tracked the new onset of renal allograft stone formation after initiating cinacalcet up to 60 mg daily, which was accompanied by persistent hypercalciuria (478.2 mg/24 h). The nephrolithiasis resolved after discontinuing cinacalcet and a subtotal parathyroidectomy. This case emphasised the significance of interval monitoring of urinary calcium excretion and imaging of the transplanted kidney in terms of the recipients treated with cinacalcet for hyperparathyroidism after renal transplantation [62]. In turn, Seikrit et al. described a case of fulminant graft failure due to extensive tubular calcinosis after initiation of a calcimimetic therapy [63].

### 3.4. Parathyreidectomy

The timing of parathyroidectomy in kidney transplant candidates suffering from secondary hyperparathyroidism before transplantation, versus early or late after transplantation, remains controversial. Kovács et al. in their publication emphasised that if the patient was already waiting for a kidney transplant, it was worth performing the parathyroid surgery prior to it [64]. Conversely, Ivarsson et al. reported that parathyroidectomy was associated with improved survival in patients on maintenance dialysis, although not in patients with a renal allograft [65]. Li et al. in their study investigated the ultrasound-guided microwave ablation (MWA) in the treatment of patients with secondary hyperparathyroidism after a renal transplantation. They demonstrated that it was a safe and effective technique for destroying parathyroid gland tissue. Furthermore, the clinical effects of this intervention were long-lasting [66]. Nevertheless, a possible dangerous consequence of parathyroidectomy on kidney transplant function was also reported. In their publication, Ferreira et al. presented a comparison between acute and long-term renal changes following total parathyroidectomy with those occurring after general or urological surgery. They found that kidney filter function decreased in the acute period after parathyroidectomy, but then it returned to normal. Renal function was therefore similar to both preoperative function and to that of a control group of kidney-transplanted patients who had undergone other general surgery procedures [67]. Chudzinski et al. also reported that in the early postoperative period following parathyroidectomy, transient reductions in graft function occurred, although the procedure did not significantly impair the transplanted kidney function. eGFR was significantly lower on days 2 to 3 compared to the pre-parathyroidectomy values (52.38 vs. 44.89 mL/min/1.73 m^2^; *p* < 0.001) [68]. In turn, Bures et al. observed no correlation between the postoperative iPTH and kidney function. They concluded that parathyroidectomy did not present negative effects on graft function, whether performed before or after (early or late) kidney transplantation [69]. Furthermore, Littbarski et al. in their study indicated that parathyroidectomy should be conducted before transplantation or after the first post-transplant year. Donor kidney function KDIGO stage III, blood group 0, and post-transplant parathyroidectomy were reported as independent significant risk factors for a compromised renal graft function in the short-term follow-up [70]. El-Husseini et al. aimed to evaluate the relationships of intraoperative iPTH with long-term iPTH levels post-parathyroidectomy in dialysis, as well as in renal transplant patients. According to their observations, intraoperative PTH after 20 min of the procedure is a good indicator of long-term PTH levels in dialysis and renal transplant patients [71]. In terms of the consequences of parathyroidectomy, it is vital to bear in mind that total parathyroidectomy increased the risk for acute hypocalcaemia after KTx, although it had no effect on long-term calcium homeostasis. Authors hypothesised that the early state of hypocalcaemia was due to the high-dose glucocorticoids required for induction, in addition to the preoperative undetectable PTH [72].

### 3.5. Etelcalcetide

A very interesting pharmacotherapeutic drug is etelcalcetide. Currently, there are no studies with this medicine in patients following kidney transplantation; however, there are results from studies in dialysis patients. Block GA et al. [73] conducted a randomized study evaluating the efficacy of etelcalcetide administered intravenously 3 times a week (n = 340), and patients were also treated with oral placebo daily. Cinacalcet was administered daily p.o. (n = 343) and placebo intravenously also 3 times a week during dialysis. The study was conducted for 26 weeks. PTH concentration reductions of more than 30% were achieved in the etelcalcetide group in 68.2% of patients vs. 57.7% for cinacalcetide. PTH concentration reductions of more than 50% were achieved in the etelcalcetide group in 52.4% of patients vs. 40.2% for cinacalcetide (*p* = 0.001). It should be noted that etelcalcetide lowered blood calcium levels more (68.9% vs. 59.8%). In another multicentre study (n = 2596), the authors also showed that etelcalcetide was effective in lowering PTH and blood calcium levels [74]. This fact highlights the important role of etelcalcetide in patients following kidney transplantation who often have tertiary hyperparathyroidism. Unfortunately, the limitation of the use of the described drug is the intravenous form of administration. No studies have investigated the use of etelcalcetid in patients following kidney transplantation but it may be useful in seriously hypercalcemic post-transplant HPT patients. Further studies are needed to evaluate clinical outcomes as well as long-term efficacy and safety.

## 4. Conclusions

In conclusion, paricalcitol, cinacalcet, and parathyroidectomy considerably decreased the iPTH level in the studied groups. Taking into consideration the analysis of the data, as well as complications occurring in the post-transplant HPT, particularly those pertaining to the cardiovascular system mainly associated with hypercalcemia, we tried to determine the best therapeutic strategy. In terms of all the analysed medications, paricalcitol most significantly increased the serum calcium concentration. In the case of active forms of vitamin D, there is a risk of developing treatment-related hypercalcemia; therefore, the use of these drugs in persistent hyperparathyroidism following kidney transplantation is questionable. Calcitriol is less effective in lowering PTH levels; yet, it causes a smaller increase in calcium levels than paricalcitol. Cinacalcet and parathyroidectomy lowered calcium levels in renal transplant patients with hypercalcaemia. In contrast, only parathyroidectomy produced no recurrence of hypercalcaemia. In the case of parathyroidectomy, very few studies with hard endpoints exist. Additionally, there is a risk of transient hypocalcaemia due to the “hungry-bone” syndrome, and persistent hypoparathyroidism with sustained hypocalcaemia/low bone turnover. More studies with regard to the safety of parathyroidectomy in renal transplant patients are necessary. Finally, cinacalcet seems to be a good candidate in the treatment of post-transplant HPT; however, it should be noted that it increases the risk of low bone turnover. Furthermore, it is vital to consider the individualisation of therapy.

In terms of the limitations of our paper, it is worth mentioning that that calcitriol was not included in Table 1 and Table 2 due to the lack of sufficient data. Moreover, we have included papers about paricalcitol in Table 2, even though some of them were based on normocalcaemic and normophosphataemic patients with mild hyperparathyroidism. Such parameters do not constitute indications for urgent treatment; nevertheless, paricalcitol alters their values. Therefore, parameters were included in the table. A significant limitation of the study is the inability to perform a meta-analysis for the described issue due to the lack of sufficient data.

## Figures and Tables

**Table 1:** Selected information with regard to the analysed studies [25,26,27,28,29,30,31,32,33,34,35,36,37].

The Active Substance	Dosage	Time Study [Weeks]	Study Group (n) * Completed	Control Group (n) * Completed	Placebo or Comparator
Paricalcitol [27]	1 μg/day and next 2 μg/day	24	22 * 20	21 * 19	other therapy
Paricalcitol [25]	2 μg/day	44	37 * 35	40	standard therapy
Paricalcitol [26]	2–3 μg/day	96	69	-	-
Paricalcitol [28]	1 μg/day	72	58	-	-
Paricalcitol [35]	1 μg/day	12	31	-	-
Paricalcitol [36]	1 μg/day	48	31	31	standard therapy
Cinacalcet [30]	30 mg/day	48	32 * 29	-	-
Cinacalcet [29]	30 mg/day	48	18	-	-
Cinacalcet [31]	30 mg/day	24	193 * 175	-	-
Cinacalcet [32]	30 mg/day	24	47 * no data	-	-
Cinacalcet [33]	30 mg/day	240	6	-	-
Cinacalcet [34]	30 mg/day	52	57 * 52	57 * 52	placebo
Cinacalcet, Parathyreidectomy [37]	30 mg every other day 30 mg/day 60 mg/day	240	15 * 13	15 * 11	parathyreidectomy

* denotes number of patients who completed the study.

**Table 2:** Changes in the serum concentration of PTH, calcium, phosphorus, and the value of albumin, or information regarding the corrected serum calcium concentration based on the albumin level [25,26,27,28,29,30,31,32,33,34,35,36,37].

The Active Substance	PTH before the Study [pg/mL]	PTH after the Study [pg/mL]	Phosphorus Serum before [mmol/L]	Phosphorus Serum after [mmol/L]	Calcium Serum before [mmol/L]	Calcium Serum after [mmol/L]	Albumin (Serum Calcium Concentrations Were Corrected for Albumin Level)
Paricalcitol [27]	115.60	63.25	1.06	1.10	2.38	2.40	no data
Paricalcitol [25]	105.62	93.36	0.93	0.95	2.37	2.39	no data
Paricalcitol [26]	288	193	1.10	1.16	2.42	2.40	no data
Paricalcitol [28]	333	181	1.10	1.13	2.32	2.40	no data
Paricalcitol [35]	216	167	1.05	1.04	2.41	2.43	no data
Paricalcitol [36]	100.00	59.70	1.00	1.01	2.33	2.36	serum calcium concentrations were corrected for the albumin level
Cinacalcet [30]	364	145	1.03	1.07	2.37	2.30	no data
Cinacalcet [29]	242.04	145.62	0.65	0.94	2.78	2.48	no data
Cinacalcet [31]	235	181	0.87	0.97	2.77	2.52	no data
Cinacalcet [32]	310.72	238.39	0.93	0.96	2.69	2.48	serum calcium concentrations were corrected for the albumin level
Cinacalcet [33]	260	237	0.90	1.01	2.74	2.56	no data
Cinacalcet [34]	327.7	169.0	0.86	1.06	2.81	2.39	serum calcium concentrations were corrected for the albumin level
Cinacalcet [37]	235.75	198.03	0.92	0.96	2.72	2.43	serum calcium concentrations were corrected for the albumin level
Parathyreidectomy [37]	348.91	47.15	0.93	1.30	2.78	2.25	

## Data Availability

Not applicable.
